# Supplementary material for: Crystal Structure and Chemical Bonds in [CuII2(Tolf)4(MeOH)2]∙2MeOH
Source: Int J Mol Sci. 2023 Jan 16;24(2):1745. doi: 10.3390/ijms24021745 (PMC9864235; doi:10.3390/ijms24021745)
Supplement: Supplementary file 1 [file ijms-24-01745-s001.zip › ijms-2104968-supplementary.pdf]

Crystal structure and chemical bonds in $[Cu^{II}_2(Tolf)_4(MeOH)_2]\cdot 2MeOH$

Irena Majerz*, Marta S. Krawczyk

Faculty of Pharmacy, Department of Fundamentals of Chemical Sciences, Wrocław Medical University, Borowska 211a, 50-556 Wrocław, Poland.

E-mail: irena.majerz@umw.edu.pl, majerz@yahoo.com

Supplementary Materials

Table S1. Geometry of C–H···π interactions in $[Cu^{II}_2(Tolf)_4(MeOH)_2]\cdot 2MeOH$ p S2

Table S2. Geometry of C–Cl···π interaction in $[Cu^{II}_2(Tolf)_4(MeOH)_2]\cdot 2MeOH$ p S2

Fig. S1. QTAIM diagram shoving C–Cl···π interactions in $[Cu^{II}_2(Tolf)_4(MeOH)_2]\cdot 2MeOH$ p S2

Fig. S2. NCI diagram shoving C–Cl···π interactions in $[Cu^{II}_2(Tolf)_4(MeOH)_2]\cdot 2MeOH$ p S3

Fig. S3. IR spectra: Top: for $[Cu^{II}_2(Tolf)_4(MeOH)_2]\cdot 2MeOH$ in the range of 4000 – 400 cm^{-1}; Down: for tolfenamic acid p S4

Fig. S4. IR spectra: Top: for $[Cu^{II}_2(Tolf)_4(MeOH)_2]\cdot 2MeOH$ in the range of 1800 – 400 cm^{-1}; Down: for tolfenamic acid p S5

Fig. S5. Raman spectrum for $Cu^{II}_2(Tolf)_4(MeOH)_2]\cdot 2MeOH$. p S6

Fig. S6.DTA (red) and DTG (blue) curves upon heating run for the $Cu^{II}_2(Tolf)_4(MeOH)_2]\cdot 2MeOH$ crystal. p S7

Fig. S7. Histograms from CSD showing the tendency of Cu(II)···Cu(II) bond lengths (in the range of the sum of van der Waals radii) in paddle-wheel-like double-core Cu(II) compounds with carboxylate ligands. **Top**: yellow plot for structures with only O-donor ligands; **Down**: orange plot – for structures with N-donor ligands apart from carboxylates. p S8

Table S1. Geometry of C–H···π interactions in [Cu^{II}_2(Tolf)$_4$(MeOH)$_2$]·2MeOH.

C–H···π	H···Cg (Å)	C···Cg (Å)	<(C–H···Cg) (°)
C2M–H2MC···Cg3	2.99	3.61	122
C11B–H11B···Cg1^{ii}	2.93	3.77	147

Symmetry code: ii = 1-x,-y,2-z

Table S2. Geometry of C–Cl···π interaction in [Cu^{II}_2(Tolf)$_4$(MeOH)$_2$]·2MeOH.

C–Cl···π	Cl···Cg (Å)	C···Cg (Å)	<(C–Cl···Cg) (°)
C10B–Cl2···Cg2^{ii}	3.43	4.86	137

Symmetry code: iii = 1-x, -y, 2-z

Centres of aromatic rings:

Cg1 [C2A/C3A/C4A/C5A/C6A/C7A],

Cg2 [C2B/C3B/C4B/C5B/C6B/C7B],

Cg3 [C8A/C9A/C10A/C11A/C12A/C13A]

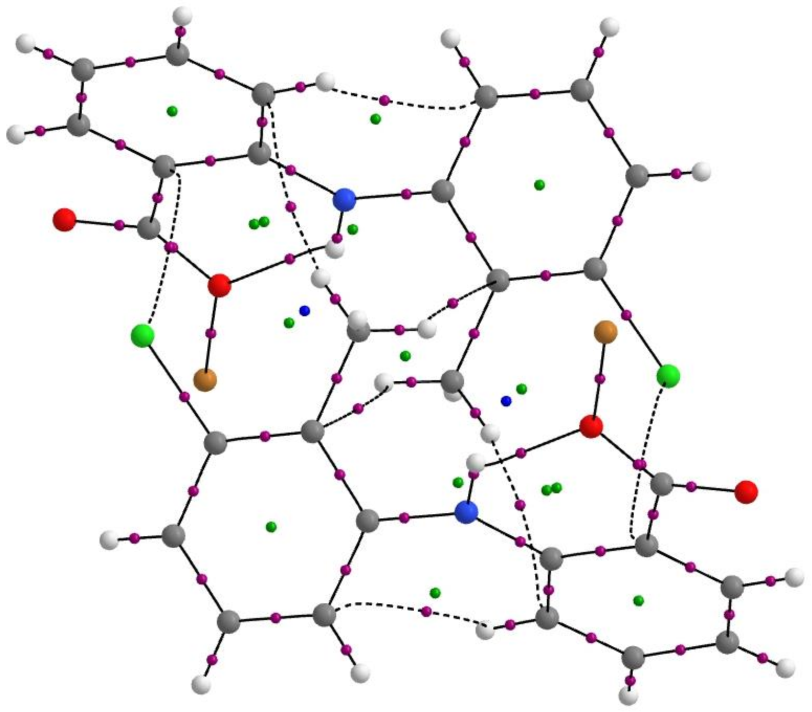

Figure S1. QTAIM diagram shoving C–Cl···π interactions in [Cu^{II}_2(Tolf)$_4$(MeOH)$_2$]·2MeOH.

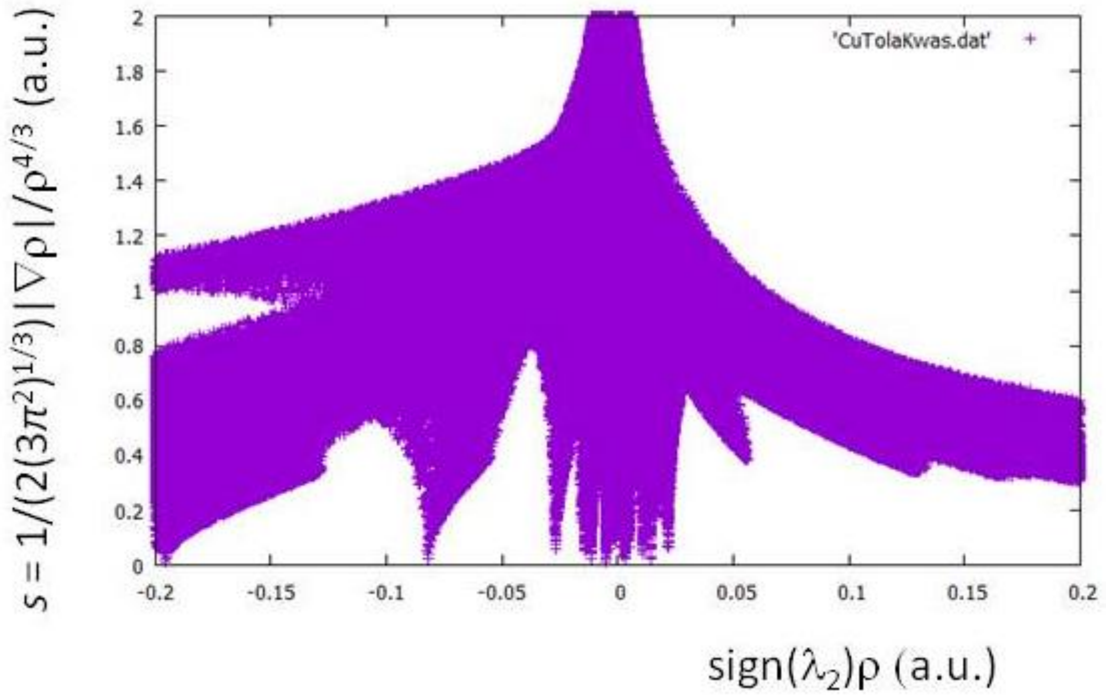

Figure S2. NCI diagram shoving C–Cl···π interactions in $[Cu^{II}_2(Tolf)_4(MeOH)_2]\cdot 2MeOH$.

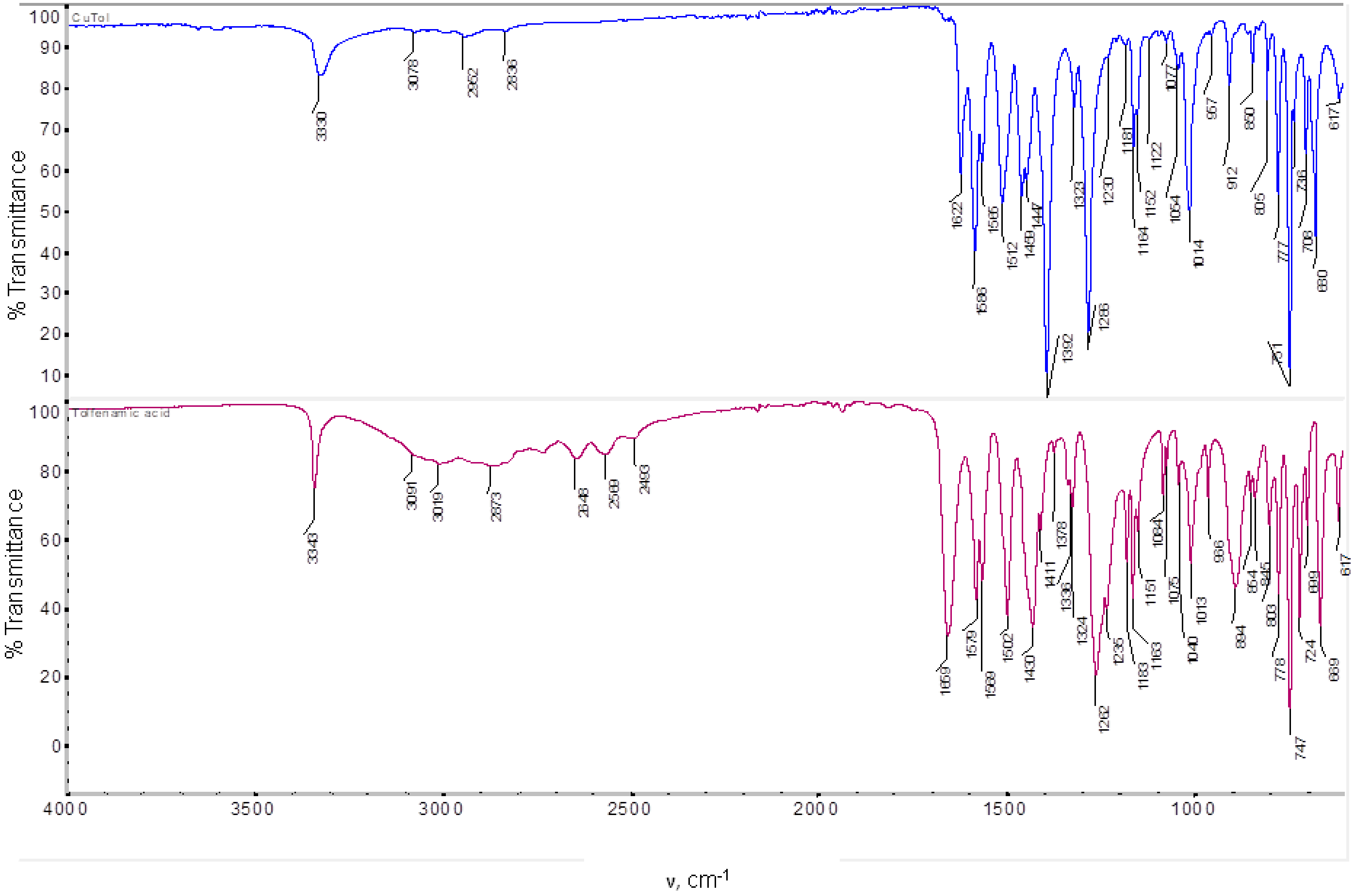

Figure S3. IR spectra: Top: for $[Cu^{II}_2(Tolf)_4(MeOH)_2]\cdot 2MeOH$ in the range of 4000 – 400 cm^{-1}; Down: for tolfenamic acid.

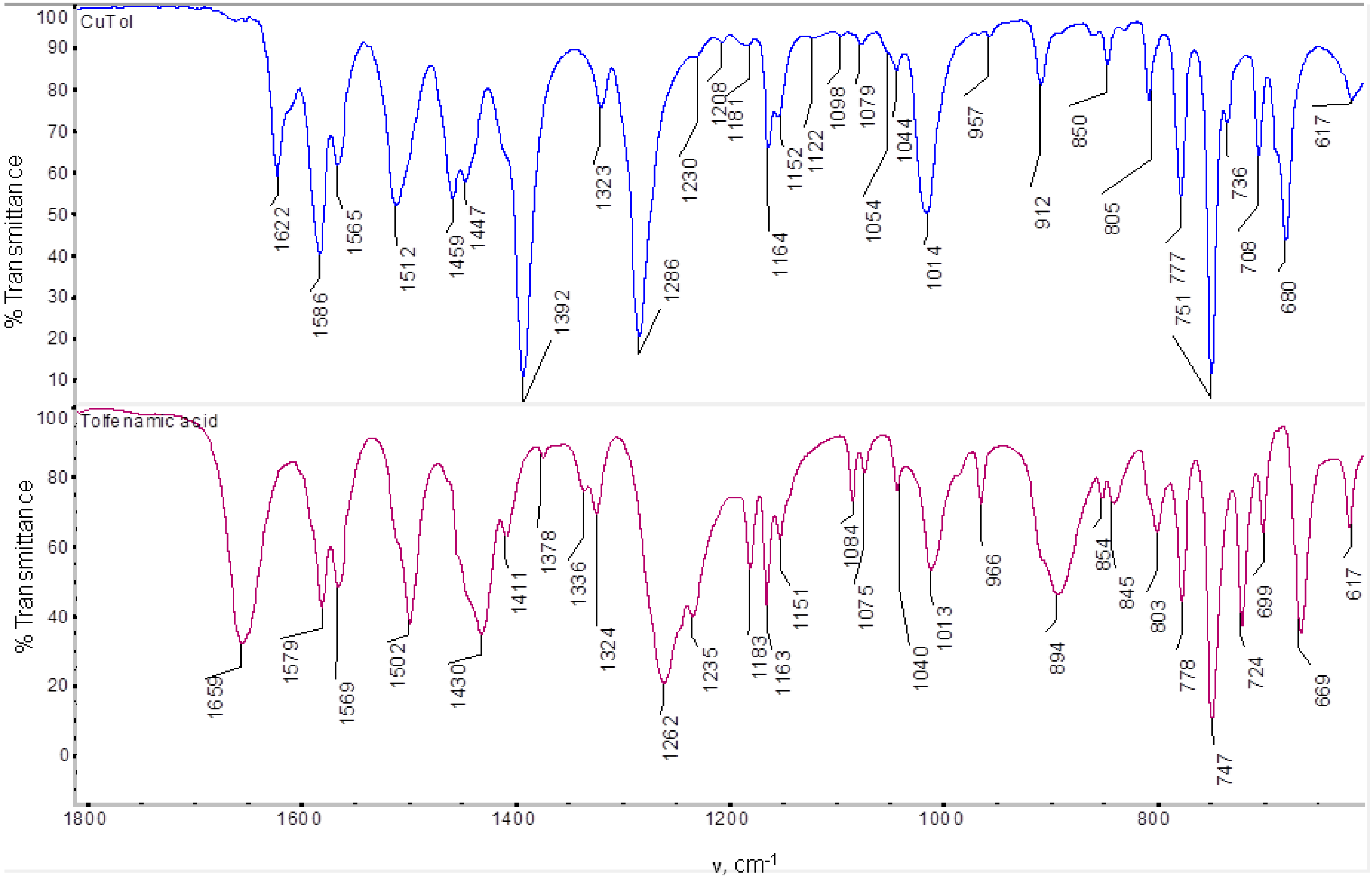

Figure S4. IR spectra: Top: for $[Cu^{II}_2(Tolf)_4(MeOH)_2]\cdot 2MeOH$ in the range of 1800 – 400 cm^{-1} Down: for tolfenamic acid.

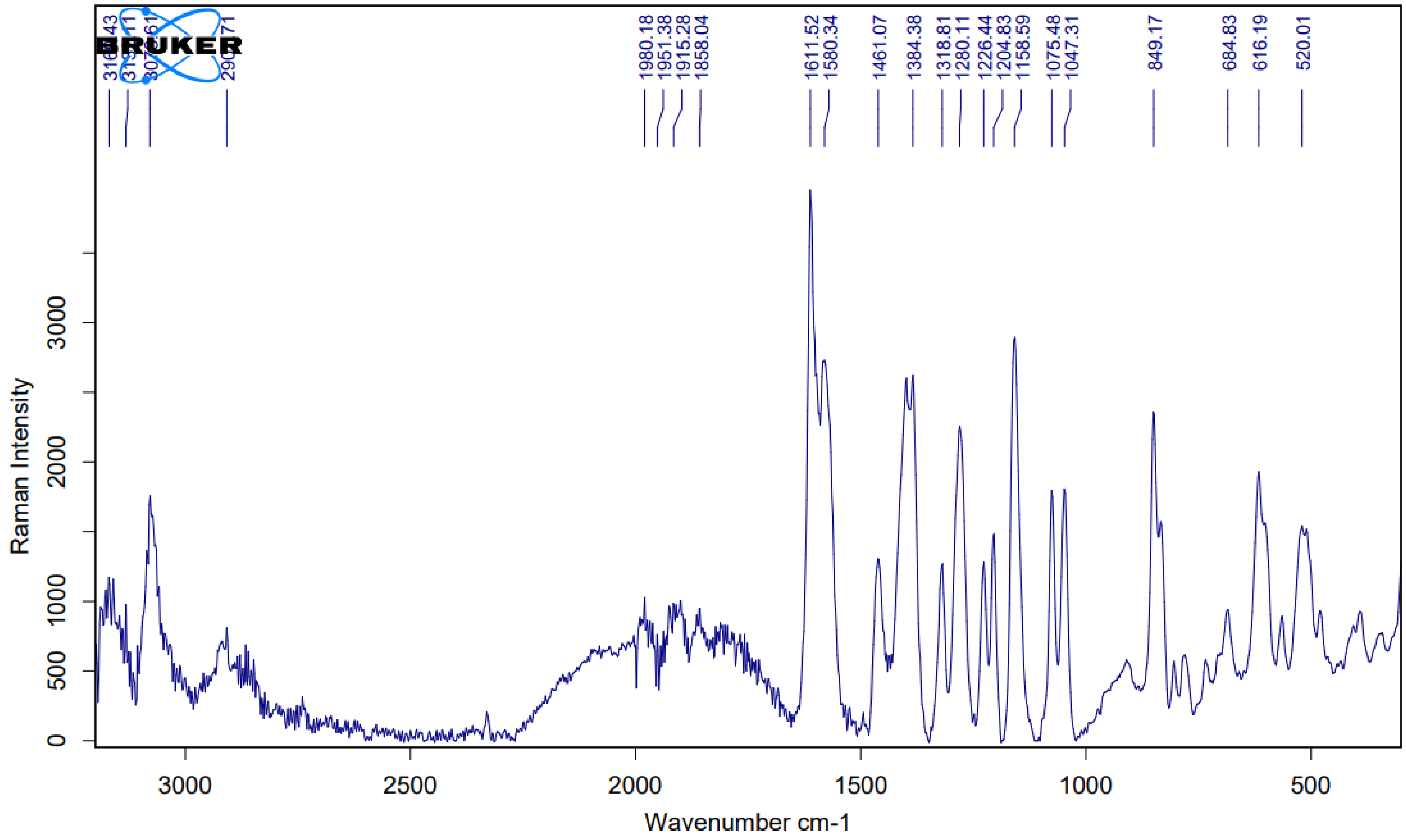

Figure S5. Raman spectrum for $[Cu^{II}_2(Tolf)_4(MeOH)_2]\cdot 2MeOH$.

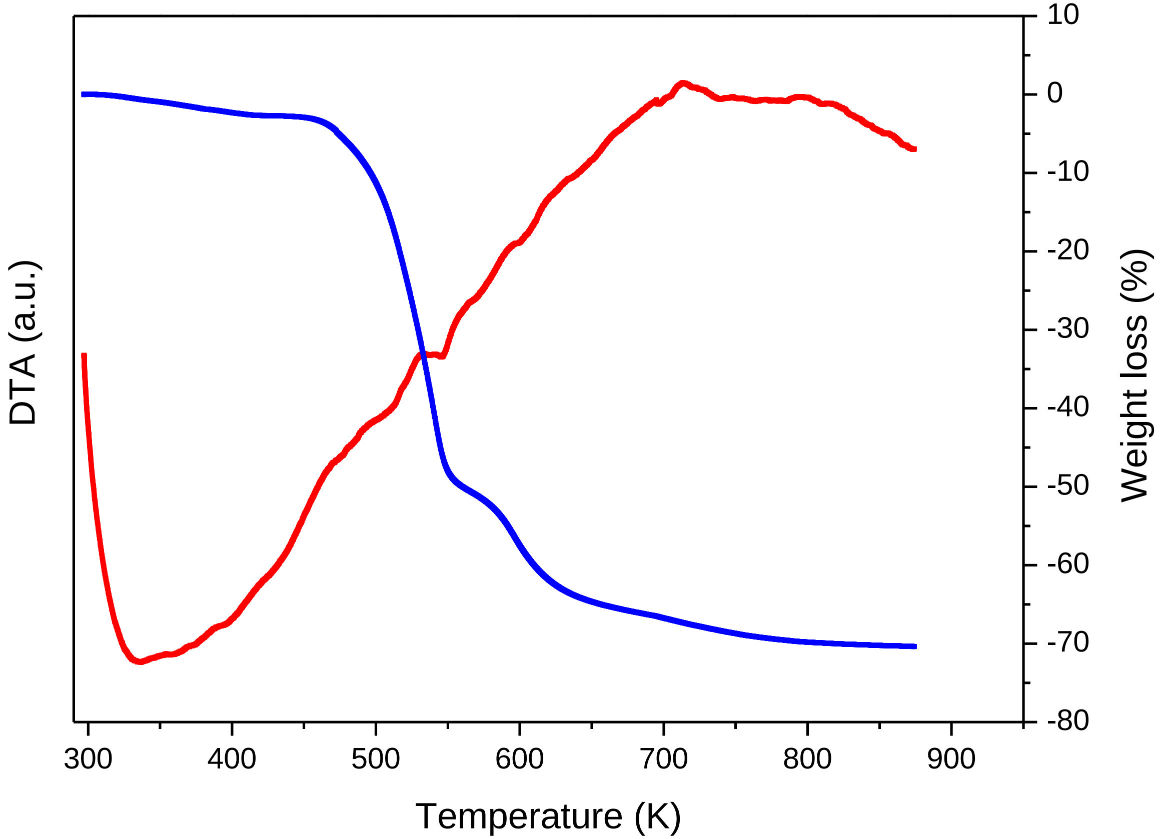

Figure S6. DTA (red) and DTG (blue) curves upon heating run for the $Cu^{II}_2(Tolf)_4(MeOH)_2]\cdot 2MeOH$ crystal.

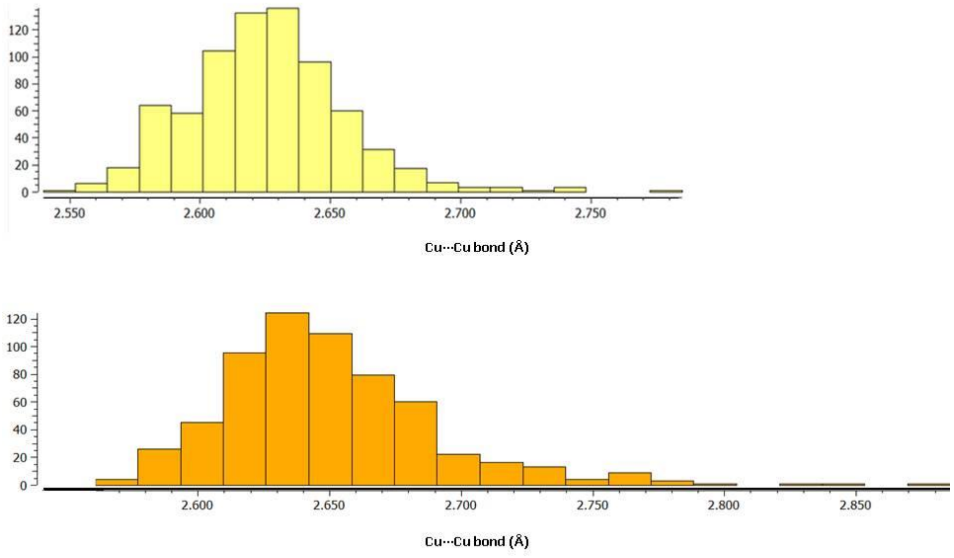

Figure S7. Histograms from CSD showing the tendency of Cu(II)···Cu(II) bond lengths (in the range of the sum of van der Waals radii) in paddle-wheel-like double-core Cu(II) compounds with carboxylate ligands. **Top**: yellow plot for structures with only O-donor ligands; **Down**: orange plot – for structures with N-donor ligands apart from carboxylates.

The adopted criterion for the distances Cu(II)···Cu(II): distances in the range of the sum of van der Waals radii, where the value of the van der Waals radius for Cu was determined by Bondi (Bondi, A. "van der Waals Volumes and Radii". *J. Phys. Chem.* **1964**, *68* (*3*) 441–451. *doi*:*10.1021/j100785a001*.)
